# Synthesis, crystal structure and photophysical properties of chlorido­[2-(2′,6′-di­fluoro-2,3′-bipyri­din-6-yl-κ*N* ^1^)-6-(pyridin-2-yl­oxy-κ*N*)phenyl-κ*C* ^1^]platinum(II)

**DOI:** 10.1107/S2056989021000128

**Published:** 2021-01-08

**Authors:** Ki-Min Park, Cheol Joo Moon, Sanghyun Paek, Youngjin Kang

**Affiliations:** a Research Institute of Natural Science, Gyeongsang National University, Jinju 52828, Republic of Korea; b Department of Chemistry & Energy Engineering, Sangmyung University, Seoul 03016, Republic of Korea; c Division of Science Education, Kangwon National University, Chuncheon 24341, Republic of Korea

**Keywords:** crystal structure, 2,3′-bi­pyridine, platinum compound, luminescence, OLED

## Abstract

The title compound, [Pt(C_21_H_12_F_2_N_3_O)Cl], crystallizes with two crystallographically independent mol­ecules (*A* and *B*) in the asymmetric unit, which adopt similar conformations. The Pt^II^ atoms in both mol­ecules adopt distorted square-planar geometries, coordinated by one C and two N atoms from the tridentate 2′,6′-di­fluoro-6-[3-(pyridin-2-yl­oxy)phen­yl]-2,3′-bi­pyridine ligand and a chloride anion: the C and Cl atoms are *trans*. In the crystal, C—H⋯Cl/F hydrogen bonds, F⋯π and weak π–π stacking inter­actions between adjacent *A* and *B* mol­ecules and between pairs of inversion-related B mol­ecules lead to the formation of a two-dimensional supra­molecular network lying parallel to the *ab* plane. The sheets are stacked along the *c-*axis direction and linked by F⋯π and weak π–π stacking inter­actions between pairs of inversion-related *A* mol­ecules, forming a three-dimensional supra­molecular network. The photoluminescence quantum efficiency of the title compound in the blue–green region of the visible region (λ_max_ = 517 and 544 nm) is estimated to be ∼0.2–0.3, indicating that the title compound could be a suitable candidate as the emitting material in organic light-emitting diode (OLED) applications.

## Abstract 2

In the crystal of the title compound, which shows blue–green emission, the mol­ecules are connected *via* C—H⋯Cl/F, halogen⋯π and weak π–π stacking inter­actions.

## Chemical context   

The C,N-chelating 2,3′-bi­pyridine-based transition-metal compounds have attracted much inter­est because of their wide applications as biological labels, photosensitizers in water reduction, sensors and organic light-emitting diodes (OLEDs) (Zaen *et al.*, 2019*a* ▸,*b* ▸). Especially, highly efficient phospho­rescent metal complexes containing Ir^III^ and Pt^II^ can be synthesized by using 2,3′-bi­pyridine as ligand, which feature a high triplet-state energy (Lee *et al.*, 2018 ▸). In terms of the efficiency and stability of OLEDs, tetra­dentate ligand-based Pt^II^ complexes are known to be very good candidates as triplet emitters (Wang & Wang, 2019 ▸). The design of tetra­dentate ligands is focused on making appropriate coordination modes in order to form five or six- membered metallacycles. To achieve blue emission in Pt-based triplet emitters, two strategies have been employed as follows: (i) incorporation of a high-triplet-energy moiety into the ligand framework; (ii) the breakage of π-conjugation in the ligand to increase the energy gap (Fleetham *et al.*, 2016 ▸; Kang *et al.*, 2020 ▸). With these in mind, we have recently synthesized 2′,6′-di­fluoro-6-[3-(pyridin-2-yl­oxy)phen­yl]-2,3′-bi­pyridine as a ligand with high triplet energy (Park *et al.*, 2020 ▸). By using this ligand, we have synthesized its coordination metal complex containing Pt^II^ and determined its crystal structure: herein, we report the structural and photophysical characteristics of chlorido­[2′,6′-di­flu­o­ro-6-[3-(pyridin-2-yl­oxy)phen­yl]-2,3′-bi­pyridine-*κ* ^3^ *N*,*C*,*N′*]platinum(II).
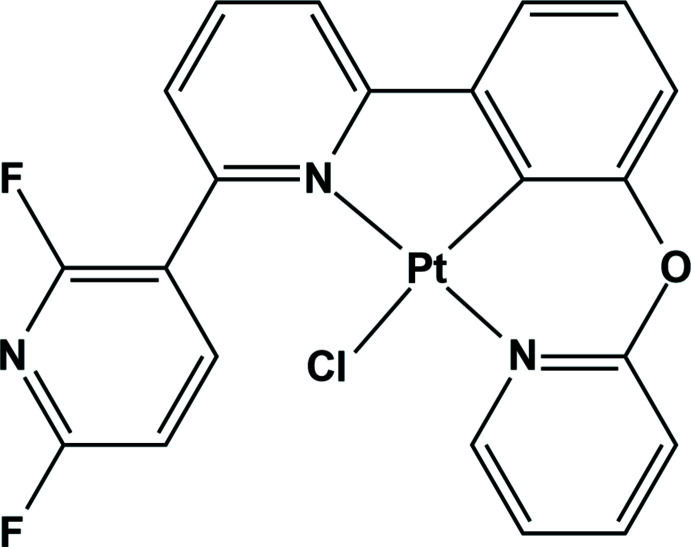


## Structural commentary   

**Figure 1:**
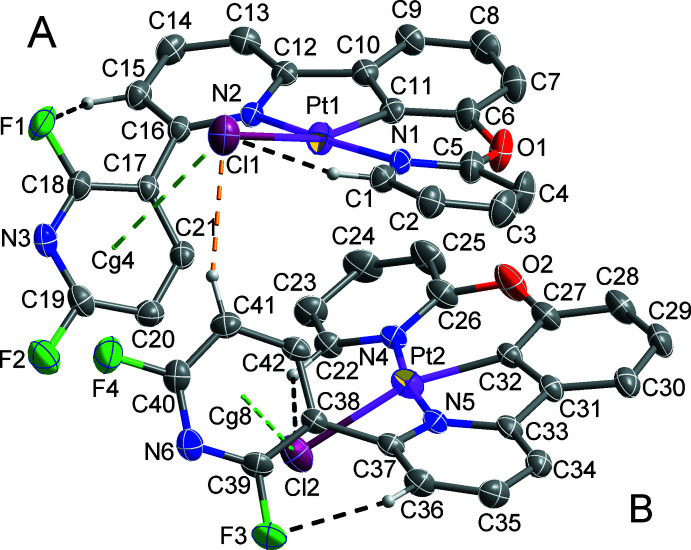
The mol­ecular structure of the title compound, showing the atom-numbering scheme and displacement ellipsoids at the 50% probability level. Black and green dashed lines represent intra­molecular C—H⋯Cl/F and Cl⋯π inter­actions, respectively, and yellow dashed line represents inter­molecular C—H⋯Cl inter­action. H atoms not involved in intra- and inter­molecular inter­actions are not shown for clarity.

The asymmetric unit of the title compound, Pt(C_21_H_12_F_2_N_3_O)Cl, contains two crystallographically independent mol­ecules (*A* and *B* denote the Pt1- and Pt2-containing mol­ecules, respectively), which adopt similar conformations (Fig. 1 ▸ and Table 1 ▸). The coordination sphere of the Pt^II^ atoms in both mol­ecules is a distorted square-planar geometry, with the respective coordination sites occupied by one C and two N atoms from the 2′,6′-di­fluoro-6-[3-(pyridin-2-yl­oxy)phen­yl]-2,3′-bi­pyridine ligand together with a chloride anion. The average length [1.949 (4) Å] of the Pt—C bonds is slightly shorter than that [2.042 (3) Å] of the Pt—N bonds because of back bonding between the metal and the anionic C atom of the ligand. The Cl1 and Cl2 atoms deviate from the mean plane consisting of the Pt and coordinated N/C atoms [r.m.s. deviations = 0.013 (1) (*A*) and 0.017 (1) Å (*B*)] with deviations of 0.700 (6) Å for *A* and 0.720 (6) Å for *B*.

In each mol­ecule, there are intra­molecular C—H⋯Cl/F inter­actions, contributing to the stabilization of the mol­ecular structure (Table 2 ▸ and black dashed lines in Fig. 1 ▸). Moreover, an intra­molecular Cl⋯π inter­action [Cl1⋯*Cg*4 = 3.4537 (19) Å, Cl2⋯*Cg*8 = 3.455 (2) Å; green dashed lines in Fig. 1 ▸; *Cg*4 and *Cg*8 are the centroids of the N3/C17–C21 and N6/C38–C42 rings, respectively] between the coordinated chloride ion and the pyridine ring with fluorine substituents are also observed. Mol­ecules *A* and *B* are inter­linked by a C—H⋯Cl inter­action (Table 2 ▸ and yellow dashed line in Fig. 1 ▸). In the 6-phenyl-2,3′-bi­pyridine system in both mol­ecules, the phenyl­pyridine moieties are approximately coplanar with the dihedral angles between the pyridine ring and the attached phenyl rings being 10.01 (11) for *A* and 9.64 (11)° for *B*. However, the terminal di­fluoro-pyridine ring is tilted by 46.08 (9) for *A* and 46.96 (8)° for *B* with respect to phenyl­pyridine ring plane. This distortion may be caused by the intra­molecular Cl⋯π inter­action described above. The pyridine ring of the pyridine-2-yl­oxy group is slightly tilted by 22.09 (13) for *A* and 19.70 (13)° for *B* relative to the phenyl­pyridine ring plane.

## Supra­molecular features   

**Figure 2:**
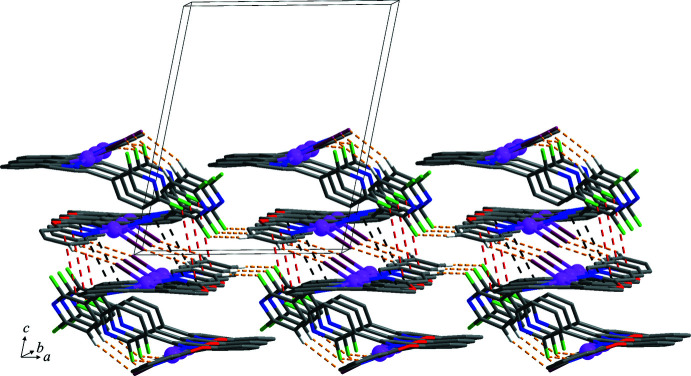
The two-dimensional supra­molecular network formed through inter­molecular C—H⋯Cl/F hydrogen bonds (yellow dashed lines), F⋯π (red dashed lines) and π–π stacking (black dashed lines) inter­actions between aromatic rings of inversion-related *B* mol­ecules. For clarity, H atoms not involved in the inter­molecular inter­actions have been omitted. Colour codes: violet = platinum, plum = chloride, green = fluorine, red = oxygen, blue = nitro­gen, grey = carbon and white = hydrogen.

**Figure 3:**
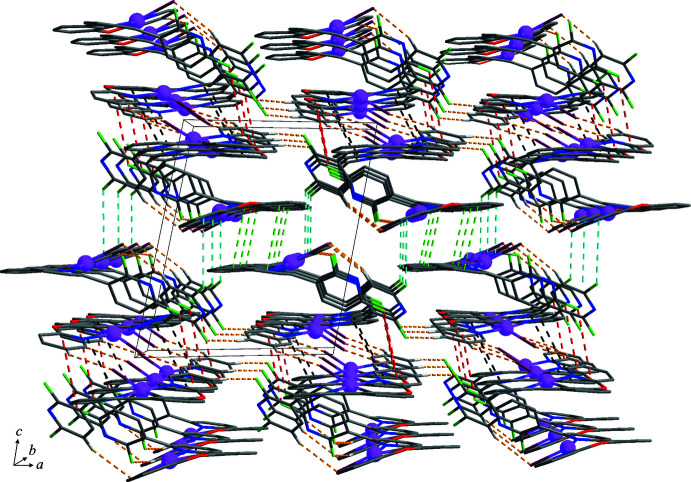
The three-dimensional supra­molecular network formed through F⋯π and π–π stacking inter­actions (sky-blue and green dashed lines) between the two-dimensional networks stacked along the *c-*axis direction. H atoms not involved in the inter­molecular inter­actions have been omitted for clarity. Colour codes: violet = platinum, plum = chloride, green = fluorine, red = oxygen, blue = nitro­gen, grey = carbon and white = hydrogen.

In the crystal structure, inter­molecular C–H⋯Cl/F hydrogen bonds (Table 2 ▸, yellow dashed lines in Figs. 1 ▸ and 2 ▸) between adjacent *A* and *B* mol­ecules and between pairs of inversion-related *B* mol­ecules lead to the formation of a two-dimensional supra­molecular network lying parallel to the *ab* plane. In addition, this network is consolidated by halogen⋯π and weak π–π stacking inter­actions [red and black dashed lines in Fig. 2 ▸, respectively; F2⋯*Cg*5^i^ = 3.819 (3); *Cg*6⋯*Cg*7^ii^ = 4.022 (2) Å; *Cg*5, *Cg*6 and *Cg*7 are the centroids of the N4/C22–C26, C27–C32 and N5/C33–C37 rings, respectively; symmetry codes: (i) −*x* + 2, −*y* + 1, −*z*; (ii) −*x* + 2, −*y* + 2, −*z*] between pairs of inversion-related *B* mol­ecules. These sheets are stacked along the *c-*axis direction and connected by F⋯π and weak π–π stacking inter­actions [sky-blue and green dashed lines in Fig. 3 ▸, respectively; F4⋯*Cg*1^iii^ = 3.834 (3) Å; *Cg*2⋯*Cg*3^iv^ = 4.073 (2) Å; *Cg*1, *Cg*2 and *Cg*3 are the centroids of the N1/C1–C5, C6–C11 and N2/C12–C16 rings, respectively; symmetry code: (iii) −*x* + 2, −*y* + 1, −*z* + 1; (iv) −*x* + 1, −*y* + 1, −*z* + 1] between pairs of inversion-related *A* mol­ecules, resulting in the formation of a three-dimensional supra­molecular network.

## Luminescent property   

**Figure 4:**
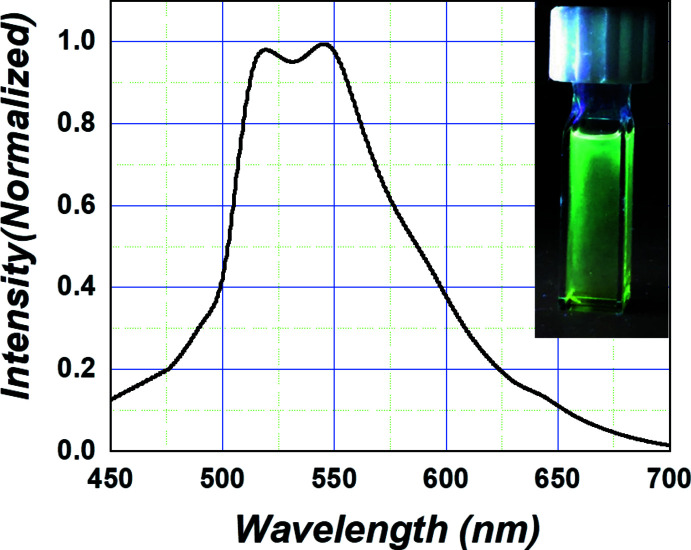
Emission spectrum of the title compound in solution at room temperature (Inset: Emission photo).

The bright blueish-green emission of the title compound in solution is dominated by phospho­rescence as supported by an excited-state lifetime of more than 1 ms. Emission maxima appear at 517 and 544 nm at room temperature, as shown in Fig. 4 ▸. The emission observed in the title compound is attributable to an intra-ligand charge transfer (ILCT) transition mixed with a metal-to-ligand charge-transfer (MLCT) transition based on previous reports (Wang & Wang, 2019 ▸). Contrary to our expectations, the title compound shows green emission. It may be that the chloride ion bound directly to the platinum ion causes this effect because 2′,6′-di­fluoro-2,3′-bi­pyridine (dfpypy)-based platinum complexes without chloride ions often exhibit blue emission at room temperature. The photoluminescence quantum efficiency of the title compound was estimated to be ∼0.2–0.3 (Fig. 4 ▸, inset). Such efficiency is enough to use the title compound as the emitting material in organic light-emitting diode (OLED) applications.

## Database survey   

A survey of *SciFinder* (SciFinder, 2020 ▸) for 6-[3-(pyridin-2-yl­oxy)phen­yl]-2,3′-bi­pyridine (*i.e*., the title ligand without specifying the fluorine substituents), gave two hits. These are the reports of the crystal structures and photophysical properties of the free ligands for 2′,6′-di­fluoro-6-[3-(pyridin-2-yl­oxy)phen­yl]-2,3′-bi­pyridine and 2′,6′-dimeth­oxy-6-[3-(pyridin-2-yl­oxy)phen­yl]-2,3′-bi­pyridine (Park *et al.*, 2020 ▸). The survey revealed no exact matches for the reported structure of the title ligand: to the best of our knowledge, this is the first crystal structure reported for a platinum complex with the title ligand.

## Synthesis and crystallization   

All experiments were performed under a dry N_2_ atmosphere using standard Schlenk techniques. All solvents used in this study were freshly distilled over appropriate drying reagents prior to use. All starting materials were commercially purchased and used without further purification. The ^1^H NMR spectrum was recorded on a JEOL 400 MHz spectrometer. The ligand, 2′,3′-di­fluoro-6-[3-(pyridin-2-yl­oxy)phen­yl]-2,3′-bi­pyridine (Park *et al.*, 2018 ▸, 2020 ▸) and starting material, PtCl_2_(PhCN)_2_, (Uchiyama *et al.*, 1980 ▸) were synthesized according to previous reports.

The title compound was synthesized as follows: A mixture of the ligand (0.36 g, 1.0 mmol), PtCl_2_(PhCN)_2_ (0.47 g, 1.0 mmol) and xylene (10 ml) was refluxed (433 K) for 48 h under an N_2_ flow. The xylene was removed by distillation and the crude product was purified by silica gel column chromatography (CH_2_Cl_2_:hexane = 1:1, *v*/*v*) to give the title compound as a yellow solid in 40% yield. Orange–red crystals suitable for X-ray crystallography analysis were obtained from a CH_2_Cl_2_/hexane solution by slow evaporation. ^1^H NMR (400 MHz, CDCl_3_) *δ* 9.91 (*dd*, *J* = 6.0, 2.0 Hz, 1H), 8.20(*m*, 1H), 7.97 (*t*, *J* = 8.0 Hz, ^1^H), 7.90–7.84 (*m*, 2H), 7.50–7.42 (*m*, 2H), 7.30–7.22 (*m*, 2H), 7.06 (*d*, *J* = 7.6 Hz, 1H), 6.97-6.91 (*m*, 2H). ^13^C NMR (100 MHz, CDCl_3_) δ 206.5, 167.3, 156.4, 155.9, 151.0, 148.1, 146.6, 146.5, 140.1, 139.0, 125.7, 125.0, 121.0, 118.4, 118.3, 117.6, 115.9, 106.0, 105.9, 105.7, 105.6. Analysis calculated for C_21_H_12_ClF_2_N_3_OPt: C 42.69; H 2.05; N 7.11%; found: C 42.70, H 2.06, N 7.09%.

## Refinement   

Crystal data, data collection and structure refinement details are summarized in Table 3 ▸. All H atoms were positioned geometrically and refined using a riding model: C—H = 0.95 Å with *U*_iso_(H) = 1.2*U*_eq_(C).

## Supplementary Material

Crystal structure: contains datablock(s) I, New_Global_Publ_Block. DOI: 10.1107/S2056989021000128/hb7960sup1.cif

Structure factors: contains datablock(s) I. DOI: 10.1107/S2056989021000128/hb7960Isup2.hkl

CCDC reference: 2053861

Additional supporting information:  crystallographic information; 3D view; checkCIF report

## Figures and Tables

**Table 1:** Selected geometric parameters (Å, °)

Pt1—C11	1.949 (4)	Pt2—C32	1.948 (4)
Pt1—N1	2.035 (3)	Pt2—N4	2.042 (3)
Pt1—N2	2.040 (3)	Pt2—N5	2.049 (3)
Pt1—Cl1	2.4193 (8)	Pt2—Cl2	2.4154 (8)
			
C11—Pt1—N1	90.92 (14)	C32—Pt2—N4	90.87 (14)
C11—Pt1—N2	80.86 (14)	C32—Pt2—N5	80.98 (13)
N1—Pt1—N2	171.61 (12)	N4—Pt2—N5	171.56 (11)
C11—Pt1—Cl1	163.28 (11)	C32—Pt2—Cl2	163.13 (11)
N1—Pt1—Cl1	94.81 (9)	N4—Pt2—Cl2	94.78 (9)
N2—Pt1—Cl1	93.56 (9)	N5—Pt2—Cl2	93.65 (8)

**Table 2:** Hydrogen-bond geometry (Å, °)

*D*—H⋯*A*	*D*—H	H⋯*A*	*D*⋯*A*	*D*—H⋯*A*
C1—H1⋯Cl1	0.95	2.46	3.175 (4)	132
C3—H3⋯F1^i^	0.95	2.53	3.474 (5)	172
C15—H15⋯F1	0.95	2.56	2.989 (5)	108
C22—H22⋯Cl2	0.95	2.45	3.170 (4)	132
C23—H23⋯Cl2^ii^	0.95	2.82	3.540 (5)	133
C24—H24⋯F3^iii^	0.95	2.50	3.443 (5)	172
C36—H36⋯F3	0.95	2.55	2.975 (5)	108
C41—H41⋯Cl1	0.95	2.79	3.684 (4)	158

Symmetry codes: (i) 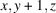; (ii) 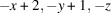; (iii) 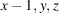.

**Table 3:** Experimental details

Crystal data	
Chemical formula	[Pt(C_21_H_12_F_2_N_3_O)Cl]
*M*_r_	590.88
Crystal system, space group	Triclinic, *P* 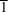
Temperature (K)	173
*a*, *b*, *c* (Å)	12.4924 (9), 12.5008 (9), 14.1163 (11)
α, β, γ (°)	74.498 (3), 73.401 (3), 61.954 (3)
*V* (Å^3^)	1841.3 (2)
*Z*	4
Radiation type	Mo *K*α
μ (mm^−1^)	7.80
Crystal size (mm)	0.46 × 0.32 × 0.24

Data collection	
Diffractometer	Bruker APEXII CCD
Absorption correction	Multi-scan (*SADABS*; Bruker, 2014 ▸)
*T*_min_, *T*_max_	0.233, 0.746
No. of measured, independent and observed [*I* > 2σ(*I*)] reflections	34679, 9126, 7667
*R*_int_	0.045
(sin θ/λ)_max_ (Å^−1^)	0.667

Refinement	
*R*[*F* ^2^ > 2σ(*F* ^2^)], *wR*(*F* ^2^), *S*	0.028, 0.067, 1.03
No. of reflections	9126
No. of parameters	523
H-atom treatment	H-atom parameters constrained
Δρ_max_, Δρ_min_ (e Å^−3^)	1.64, −1.21

Computer programs: *APEX2* (Bruker, 2014 ▸), *SAINT* (Bruker, 2014 ▸), *SHELXS97* (Sheldrick, 2008 ▸), *SHELXL2014/7* (Sheldrick, 2015 ▸), *DIAMOND* (Brandenburg, 2010 ▸), *SHELXTL* (Sheldrick, 2008 ▸) and *publCIF* (Westrip, 2010 ▸).

## supplementary crystallographic information

### Crystal data

**Table:**

[Pt(C_21_H_12_F_2_N_3_O)Cl]	*Z* = 4
*M**_r_* = 590.88	*F*(000) = 1120
Triclinic, *P*1	*D*_x_ = 2.131 Mg m^−^^3^
*a* = 12.4924 (9) Å	Mo *K*α radiation, λ = 0.71073 Å
*b* = 12.5008 (9) Å	Cell parameters from 9258 reflections
*c* = 14.1163 (11) Å	θ = 2.6–28.3°
α = 74.498 (3)°	µ = 7.80 mm^−^^1^
β = 73.401 (3)°	*T* = 173 K
γ = 61.954 (3)°	Block, yellow
*V* = 1841.3 (2) Å^3^	0.46 × 0.32 × 0.24 mm

### Data collection

**Table:**

Bruker APEXII CCD diffractometer	7667 reflections with *I* > 2σ(*I*)
φ and ω scans	*R*_int_ = 0.045
Absorption correction: multi-scan (SADABS; Bruker, 2014)	θ_max_ = 28.3°, θ_min_ = 1.5°
*T*_min_ = 0.233, *T*_max_ = 0.746	*h* = −16→16
34679 measured reflections	*k* = −15→16
9126 independent reflections	*l* = −18→18

### Refinement

**Table:**

Refinement on *F*^2^	0 restraints
Least-squares matrix: full	Hydrogen site location: inferred from neighbouring sites
*R*[*F*^2^ > 2σ(*F*^2^)] = 0.028	H-atom parameters constrained
*wR*(*F*^2^) = 0.067	*w* = 1/[σ^2^(*F*_o_^2^) + (0.0327*P*)^2^] where *P* = (*F*_o_^2^ + 2*F*_c_^2^)/3
*S* = 1.03	(Δ/σ)_max_ = 0.001
9126 reflections	Δρ_max_ = 1.64 e Å^−^^3^
523 parameters	Δρ_min_ = −1.21 e Å^−^^3^

### Special details

**Table:**

Geometry. All esds (except the esd in the dihedral angle between two l.s. planes) are estimated using the full covariance matrix. The cell esds are taken into account individually in the estimation of esds in distances, angles and torsion angles; correlations between esds in cell parameters are only used when they are defined by crystal symmetry. An approximate (isotropic) treatment of cell esds is used for estimating esds involving l.s. planes.

### Fractional atomic coordinates and isotropic or equivalent isotropic displacement parameters (Å^2^)

**Table:**

	*x*	*y*	*z*	*U*_iso_*/*U*_eq_	
Pt1	0.69646 (2)	0.61157 (2)	0.39509 (2)	0.03247 (5)	
Pt2	0.88563 (2)	0.80611 (2)	0.10492 (2)	0.03240 (5)	
Cl1	0.86494 (8)	0.44909 (8)	0.47237 (7)	0.0410 (2)	
Cl2	1.05014 (8)	0.63393 (8)	0.02853 (7)	0.0413 (2)	
F1	0.8999 (2)	0.1430 (2)	0.4219 (2)	0.0689 (7)	
F2	1.2132 (2)	0.1920 (3)	0.1978 (2)	0.0730 (8)	
F3	1.3562 (2)	0.6034 (2)	0.0774 (2)	0.0685 (7)	
F4	1.3006 (3)	0.2977 (2)	0.3009 (2)	0.0711 (7)	
O1	0.5471 (3)	0.9163 (2)	0.3614 (3)	0.0668 (9)	
O2	0.5792 (2)	0.9597 (3)	0.1333 (3)	0.0674 (9)	
N1	0.7262 (3)	0.7497 (3)	0.4159 (2)	0.0358 (7)	
N2	0.6446 (3)	0.4930 (3)	0.3661 (2)	0.0340 (7)	
N3	1.0540 (3)	0.1720 (3)	0.3099 (3)	0.0504 (9)	
N4	0.7472 (3)	0.7748 (3)	0.0861 (2)	0.0358 (7)	
N5	1.0042 (3)	0.8593 (3)	0.1331 (2)	0.0340 (7)	
N6	1.3250 (3)	0.4527 (3)	0.1888 (3)	0.0479 (8)	
C1	0.8297 (4)	0.7229 (4)	0.4491 (3)	0.0421 (9)	
H1	0.8809	0.6389	0.4688	0.050*	
C2	0.8652 (5)	0.8078 (4)	0.4562 (4)	0.0550 (11)	
H2	0.9387	0.7833	0.4797	0.066*	
C3	0.7916 (5)	0.9306 (4)	0.4283 (4)	0.0661 (13)	
H3	0.8138	0.9921	0.4322	0.079*	
C4	0.6870 (5)	0.9618 (4)	0.3953 (4)	0.0657 (13)	
H4	0.6358	1.0455	0.3748	0.079*	
C5	0.6559 (4)	0.8701 (4)	0.3920 (3)	0.0511 (11)	
C6	0.4800 (4)	0.8536 (4)	0.3637 (3)	0.0505 (10)	
C7	0.3573 (4)	0.9249 (4)	0.3571 (3)	0.0586 (12)	
H7	0.3244	1.0117	0.3499	0.070*	
C8	0.2835 (4)	0.8706 (4)	0.3610 (3)	0.0587 (12)	
H8	0.1985	0.9199	0.3585	0.070*	
C9	0.3308 (4)	0.7440 (4)	0.3685 (3)	0.0490 (10)	
H9	0.2791	0.7059	0.3720	0.059*	
C10	0.4556 (4)	0.6748 (4)	0.3706 (3)	0.0416 (9)	
C11	0.5324 (4)	0.7272 (3)	0.3681 (3)	0.0393 (8)	
C12	0.5184 (3)	0.5402 (3)	0.3718 (3)	0.0375 (8)	
C13	0.4631 (4)	0.4677 (4)	0.3732 (3)	0.0459 (10)	
H13	0.3762	0.5021	0.3785	0.055*	
C14	0.5330 (4)	0.3454 (4)	0.3668 (3)	0.0498 (10)	
H14	0.4949	0.2933	0.3719	0.060*	
C15	0.6593 (4)	0.2998 (4)	0.3530 (3)	0.0463 (9)	
H15	0.7091	0.2161	0.3457	0.056*	
C16	0.7148 (4)	0.3742 (3)	0.3495 (3)	0.0370 (8)	
C17	0.8482 (3)	0.3332 (3)	0.3148 (3)	0.0365 (8)	
C18	0.9353 (4)	0.2176 (3)	0.3469 (3)	0.0461 (10)	
C19	1.0912 (4)	0.2433 (4)	0.2383 (3)	0.0492 (10)	
C20	1.0203 (4)	0.3602 (4)	0.1990 (3)	0.0475 (9)	
H20	1.0544	0.4082	0.1473	0.057*	
C21	0.8959 (4)	0.4040 (3)	0.2393 (3)	0.0426 (9)	
H21	0.8418	0.4847	0.2146	0.051*	
C22	0.7757 (3)	0.6672 (4)	0.0570 (3)	0.0408 (9)	
H22	0.8606	0.6136	0.0403	0.049*	
C23	0.6911 (4)	0.6315 (5)	0.0502 (4)	0.0566 (12)	
H23	0.7162	0.5556	0.0294	0.068*	
C24	0.5660 (4)	0.7100 (5)	0.0748 (4)	0.0658 (14)	
H24	0.5042	0.6880	0.0715	0.079*	
C25	0.5344 (4)	0.8173 (5)	0.1032 (4)	0.0655 (13)	
H25	0.4499	0.8718	0.1202	0.079*	
C26	0.6258 (4)	0.8477 (4)	0.1076 (3)	0.0500 (10)	
C27	0.6426 (4)	1.0252 (4)	0.1338 (3)	0.0480 (10)	
C28	0.5712 (4)	1.1484 (4)	0.1399 (3)	0.0576 (12)	
H28	0.4841	1.1823	0.1463	0.069*	
C29	0.6266 (4)	1.2206 (4)	0.1368 (3)	0.0589 (12)	
H29	0.5775	1.3056	0.1394	0.071*	
C30	0.7530 (4)	1.1732 (4)	0.1298 (3)	0.0507 (10)	
H30	0.7910	1.2249	0.1261	0.061*	
C31	0.8231 (4)	1.0476 (4)	0.1284 (3)	0.0398 (9)	
C32	0.7694 (3)	0.9716 (3)	0.1304 (3)	0.0380 (8)	
C33	0.9573 (4)	0.9852 (4)	0.1276 (3)	0.0398 (9)	
C34	1.0291 (4)	1.0404 (4)	0.1256 (3)	0.0439 (9)	
H34	0.9949	1.1272	0.1193	0.053*	
C35	1.1513 (4)	0.9709 (4)	0.1327 (3)	0.0499 (10)	
H35	1.2035	1.0087	0.1281	0.060*	
C36	1.1962 (4)	0.8450 (4)	0.1465 (3)	0.0466 (9)	
H36	1.2797	0.7955	0.1541	0.056*	
C37	1.1221 (3)	0.7895 (4)	0.1496 (3)	0.0370 (8)	
C38	1.1627 (3)	0.6569 (3)	0.1841 (3)	0.0349 (8)	
C39	1.2793 (3)	0.5693 (4)	0.1529 (3)	0.0452 (10)	
C40	1.2510 (4)	0.4174 (4)	0.2611 (3)	0.0494 (10)	
C41	1.1313 (4)	0.4895 (4)	0.2994 (3)	0.0463 (9)	
H41	1.0810	0.4570	0.3502	0.056*	
C42	1.0885 (3)	0.6118 (4)	0.2596 (3)	0.0414 (9)	
H42	1.0066	0.6665	0.2843	0.050*	

### Atomic displacement parameters (Å^2^)

**Table:**

	*U*^11^	*U*^22^	*U*^33^	*U*^12^	*U*^13^	*U*^23^
Pt1	0.03777 (9)	0.02938 (8)	0.03200 (8)	−0.01344 (6)	−0.01039 (6)	−0.00506 (6)
Pt2	0.02768 (8)	0.03840 (9)	0.03250 (8)	−0.01276 (6)	−0.00509 (6)	−0.01087 (6)
Cl1	0.0438 (5)	0.0350 (4)	0.0486 (5)	−0.0142 (4)	−0.0198 (4)	−0.0065 (4)
Cl2	0.0340 (5)	0.0435 (5)	0.0498 (5)	−0.0123 (4)	−0.0076 (4)	−0.0212 (4)
F1	0.0752 (18)	0.0410 (13)	0.0822 (19)	−0.0289 (13)	−0.0188 (15)	0.0153 (13)
F2	0.0478 (15)	0.0675 (18)	0.093 (2)	−0.0155 (14)	−0.0027 (14)	−0.0253 (15)
F3	0.0387 (13)	0.0711 (17)	0.0841 (19)	−0.0261 (13)	0.0130 (12)	−0.0154 (14)
F4	0.0702 (18)	0.0477 (15)	0.0856 (19)	−0.0189 (14)	−0.0214 (15)	−0.0003 (14)
O1	0.071 (2)	0.0312 (15)	0.105 (3)	−0.0161 (15)	−0.047 (2)	0.0014 (16)
O2	0.0295 (15)	0.071 (2)	0.104 (3)	−0.0123 (15)	−0.0051 (15)	−0.0447 (19)
N1	0.0453 (18)	0.0280 (15)	0.0366 (16)	−0.0167 (14)	−0.0065 (13)	−0.0082 (12)
N2	0.0411 (17)	0.0318 (16)	0.0313 (16)	−0.0158 (14)	−0.0099 (13)	−0.0047 (12)
N3	0.050 (2)	0.0360 (18)	0.067 (2)	−0.0129 (17)	−0.0152 (18)	−0.0159 (17)
N4	0.0288 (15)	0.0432 (18)	0.0364 (16)	−0.0142 (14)	−0.0073 (12)	−0.0088 (14)
N5	0.0321 (16)	0.0387 (17)	0.0330 (16)	−0.0136 (14)	−0.0054 (12)	−0.0122 (13)
N6	0.0372 (18)	0.045 (2)	0.061 (2)	−0.0130 (16)	−0.0155 (17)	−0.0106 (17)
C1	0.048 (2)	0.042 (2)	0.045 (2)	−0.0228 (19)	−0.0103 (18)	−0.0106 (17)
C2	0.064 (3)	0.051 (3)	0.062 (3)	−0.030 (2)	−0.014 (2)	−0.015 (2)
C3	0.090 (4)	0.049 (3)	0.079 (3)	−0.040 (3)	−0.024 (3)	−0.012 (2)
C4	0.084 (3)	0.031 (2)	0.092 (4)	−0.022 (2)	−0.039 (3)	−0.006 (2)
C5	0.062 (3)	0.037 (2)	0.052 (3)	−0.015 (2)	−0.018 (2)	−0.0069 (19)
C6	0.056 (3)	0.037 (2)	0.053 (3)	−0.011 (2)	−0.021 (2)	−0.0040 (19)
C7	0.063 (3)	0.041 (2)	0.066 (3)	−0.006 (2)	−0.031 (2)	−0.008 (2)
C8	0.046 (2)	0.061 (3)	0.057 (3)	−0.008 (2)	−0.022 (2)	−0.006 (2)
C9	0.041 (2)	0.056 (3)	0.047 (2)	−0.011 (2)	−0.0166 (18)	−0.011 (2)
C10	0.045 (2)	0.045 (2)	0.034 (2)	−0.0149 (19)	−0.0134 (17)	−0.0064 (17)
C11	0.044 (2)	0.038 (2)	0.0312 (19)	−0.0135 (18)	−0.0126 (16)	−0.0006 (16)
C12	0.041 (2)	0.042 (2)	0.0297 (19)	−0.0163 (18)	−0.0118 (16)	−0.0026 (16)
C13	0.045 (2)	0.061 (3)	0.041 (2)	−0.025 (2)	−0.0128 (18)	−0.0124 (19)
C14	0.063 (3)	0.054 (3)	0.052 (2)	−0.038 (2)	−0.018 (2)	−0.007 (2)
C15	0.058 (3)	0.042 (2)	0.046 (2)	−0.025 (2)	−0.0131 (19)	−0.0092 (18)
C16	0.049 (2)	0.0316 (18)	0.0343 (19)	−0.0173 (17)	−0.0125 (16)	−0.0048 (15)
C17	0.045 (2)	0.0310 (18)	0.037 (2)	−0.0149 (17)	−0.0091 (16)	−0.0110 (16)
C18	0.055 (3)	0.034 (2)	0.053 (3)	−0.020 (2)	−0.012 (2)	−0.0076 (18)
C19	0.043 (2)	0.047 (2)	0.058 (3)	−0.013 (2)	−0.010 (2)	−0.022 (2)
C20	0.052 (2)	0.052 (2)	0.041 (2)	−0.026 (2)	−0.0040 (18)	−0.0090 (19)
C21	0.054 (2)	0.036 (2)	0.035 (2)	−0.0141 (18)	−0.0130 (17)	−0.0053 (16)
C22	0.0339 (19)	0.048 (2)	0.047 (2)	−0.0187 (18)	−0.0086 (16)	−0.0132 (18)
C23	0.051 (3)	0.067 (3)	0.065 (3)	−0.033 (2)	−0.011 (2)	−0.016 (2)
C24	0.042 (3)	0.094 (4)	0.081 (3)	−0.039 (3)	−0.010 (2)	−0.027 (3)
C25	0.032 (2)	0.081 (3)	0.091 (4)	−0.020 (2)	−0.008 (2)	−0.038 (3)
C26	0.036 (2)	0.064 (3)	0.052 (3)	−0.017 (2)	−0.0089 (18)	−0.020 (2)
C27	0.036 (2)	0.055 (3)	0.048 (2)	−0.011 (2)	−0.0038 (18)	−0.021 (2)
C28	0.039 (2)	0.063 (3)	0.064 (3)	−0.004 (2)	−0.011 (2)	−0.032 (2)
C29	0.055 (3)	0.045 (2)	0.060 (3)	−0.001 (2)	−0.010 (2)	−0.024 (2)
C30	0.058 (3)	0.044 (2)	0.045 (2)	−0.013 (2)	−0.008 (2)	−0.0184 (19)
C31	0.040 (2)	0.040 (2)	0.037 (2)	−0.0118 (18)	−0.0059 (16)	−0.0147 (17)
C32	0.035 (2)	0.042 (2)	0.0330 (19)	−0.0118 (17)	−0.0047 (15)	−0.0111 (16)
C33	0.042 (2)	0.043 (2)	0.033 (2)	−0.0153 (18)	0.0004 (16)	−0.0188 (17)
C34	0.052 (2)	0.042 (2)	0.040 (2)	−0.019 (2)	−0.0089 (18)	−0.0125 (17)
C35	0.053 (3)	0.060 (3)	0.055 (3)	−0.035 (2)	−0.0053 (19)	−0.019 (2)
C36	0.036 (2)	0.058 (3)	0.052 (2)	−0.021 (2)	−0.0093 (17)	−0.015 (2)
C37	0.036 (2)	0.047 (2)	0.0302 (19)	−0.0164 (18)	−0.0043 (15)	−0.0132 (16)
C38	0.0292 (18)	0.044 (2)	0.0349 (19)	−0.0143 (16)	−0.0083 (15)	−0.0107 (16)
C39	0.032 (2)	0.062 (3)	0.047 (2)	−0.025 (2)	−0.0054 (17)	−0.011 (2)
C40	0.050 (2)	0.045 (2)	0.058 (3)	−0.017 (2)	−0.022 (2)	−0.008 (2)
C41	0.050 (2)	0.055 (3)	0.035 (2)	−0.025 (2)	−0.0102 (18)	−0.0031 (18)
C42	0.035 (2)	0.053 (2)	0.034 (2)	−0.0157 (18)	−0.0054 (15)	−0.0114 (17)

### Geometric parameters (Å, º)

**Table:**

Pt1—C11	1.949 (4)	C12—C13	1.368 (5)
Pt1—N1	2.035 (3)	C13—C14	1.370 (6)
Pt1—N2	2.040 (3)	C13—H13	0.9500
Pt1—Cl1	2.4193 (8)	C14—C15	1.375 (6)
Pt2—C32	1.948 (4)	C14—H14	0.9500
Pt2—N4	2.042 (3)	C15—C16	1.381 (5)
Pt2—N5	2.049 (3)	C15—H15	0.9500
Pt2—Cl2	2.4154 (8)	C16—C17	1.465 (5)
F1—C18	1.334 (5)	C17—C21	1.380 (5)
F2—C19	1.356 (5)	C17—C18	1.389 (5)
F3—C39	1.341 (4)	C19—C20	1.363 (6)
F4—C40	1.347 (5)	C20—C21	1.379 (5)
O1—C5	1.354 (5)	C20—H20	0.9500
O1—C6	1.380 (5)	C21—H21	0.9500
O2—C26	1.347 (5)	C22—C23	1.360 (6)
O2—C27	1.385 (5)	C22—H22	0.9500
N1—C5	1.344 (5)	C23—C24	1.398 (6)
N1—C1	1.364 (5)	C23—H23	0.9500
N2—C16	1.369 (5)	C24—C25	1.348 (7)
N2—C12	1.385 (5)	C24—H24	0.9500
N3—C19	1.292 (6)	C25—C26	1.382 (6)
N3—C18	1.311 (5)	C25—H25	0.9500
N4—C26	1.344 (5)	C27—C28	1.382 (6)
N4—C22	1.366 (5)	C27—C32	1.391 (5)
N5—C37	1.365 (5)	C28—C29	1.357 (7)
N5—C33	1.386 (5)	C28—H28	0.9500
N6—C40	1.301 (6)	C29—C30	1.384 (6)
N6—C39	1.302 (5)	C29—H29	0.9500
C1—C2	1.364 (6)	C30—C31	1.395 (5)
C1—H1	0.9500	C30—H30	0.9500
C2—C3	1.383 (6)	C31—C32	1.389 (5)
C2—H2	0.9500	C31—C33	1.477 (5)
C3—C4	1.359 (7)	C33—C34	1.355 (5)
C3—H3	0.9500	C34—C35	1.374 (6)
C4—C5	1.388 (6)	C34—H34	0.9500
C4—H4	0.9500	C35—C36	1.378 (6)
C6—C7	1.380 (6)	C35—H35	0.9500
C6—C11	1.388 (5)	C36—C37	1.381 (5)
C7—C8	1.362 (7)	C36—H36	0.9500
C7—H7	0.9500	C37—C38	1.470 (5)
C8—C9	1.391 (6)	C38—C39	1.380 (5)
C8—H8	0.9500	C38—C42	1.386 (5)
C9—C10	1.387 (5)	C40—C41	1.369 (6)
C9—H9	0.9500	C41—C42	1.371 (5)
C10—C11	1.383 (6)	C41—H41	0.9500
C10—C12	1.482 (5)	C42—H42	0.9500
			
C11—Pt1—N1	90.92 (14)	C21—C17—C16	121.3 (3)
C11—Pt1—N2	80.86 (14)	C18—C17—C16	123.7 (4)
N1—Pt1—N2	171.61 (12)	N3—C18—F1	114.5 (4)
C11—Pt1—Cl1	163.28 (11)	N3—C18—C17	125.7 (4)
N1—Pt1—Cl1	94.81 (9)	F1—C18—C17	119.7 (4)
N2—Pt1—Cl1	93.56 (9)	N3—C19—F2	114.7 (4)
C32—Pt2—N4	90.87 (14)	N3—C19—C20	126.8 (4)
C32—Pt2—N5	80.98 (13)	F2—C19—C20	118.4 (4)
N4—Pt2—N5	171.56 (11)	C19—C20—C21	115.4 (4)
C32—Pt2—Cl2	163.13 (11)	C19—C20—H20	122.3
N4—Pt2—Cl2	94.78 (9)	C21—C20—H20	122.3
N5—Pt2—Cl2	93.65 (8)	C20—C21—C17	121.5 (4)
C5—O1—C6	127.3 (3)	C20—C21—H21	119.3
C26—O2—C27	128.0 (3)	C17—C21—H21	119.3
C5—N1—C1	114.8 (3)	C23—C22—N4	124.6 (4)
C5—N1—Pt1	125.2 (3)	C23—C22—H22	117.7
C1—N1—Pt1	119.8 (2)	N4—C22—H22	117.7
C16—N2—C12	117.5 (3)	C22—C23—C24	118.0 (4)
C16—N2—Pt1	129.2 (3)	C22—C23—H23	121.0
C12—N2—Pt1	113.1 (2)	C24—C23—H23	121.0
C19—N3—C18	115.8 (4)	C25—C24—C23	119.1 (4)
C26—N4—C22	115.1 (3)	C25—C24—H24	120.5
C26—N4—Pt2	125.5 (3)	C23—C24—H24	120.5
C22—N4—Pt2	119.2 (2)	C24—C25—C26	119.6 (4)
C37—N5—C33	117.7 (3)	C24—C25—H25	120.2
C37—N5—Pt2	129.0 (2)	C26—C25—H25	120.2
C33—N5—Pt2	113.1 (2)	N4—C26—O2	124.0 (4)
C40—N6—C39	115.3 (4)	N4—C26—C25	123.7 (4)
C2—C1—N1	124.8 (4)	O2—C26—C25	112.2 (4)
C2—C1—H1	117.6	C28—C27—O2	115.6 (4)
N1—C1—H1	117.6	C28—C27—C32	121.5 (4)
C1—C2—C3	118.4 (4)	O2—C27—C32	122.9 (4)
C1—C2—H2	120.8	C29—C28—C27	119.5 (4)
C3—C2—H2	120.8	C29—C28—H28	120.3
C4—C3—C2	118.9 (4)	C27—C28—H28	120.3
C4—C3—H3	120.5	C28—C29—C30	121.5 (4)
C2—C3—H3	120.5	C28—C29—H29	119.2
C3—C4—C5	119.3 (4)	C30—C29—H29	119.2
C3—C4—H4	120.4	C29—C30—C31	118.4 (4)
C5—C4—H4	120.4	C29—C30—H30	120.8
N1—C5—O1	124.2 (4)	C31—C30—H30	120.8
N1—C5—C4	123.8 (4)	C32—C31—C30	121.4 (4)
O1—C5—C4	112.0 (4)	C32—C31—C33	115.2 (3)
O1—C6—C7	115.9 (4)	C30—C31—C33	123.3 (4)
O1—C6—C11	122.8 (4)	C31—C32—C27	117.6 (4)
C7—C6—C11	121.3 (4)	C31—C32—Pt2	114.9 (3)
C8—C7—C6	119.9 (4)	C27—C32—Pt2	126.6 (3)
C8—C7—H7	120.1	C34—C33—N5	122.0 (4)
C6—C7—H7	120.1	C34—C33—C31	125.6 (4)
C7—C8—C9	120.9 (4)	N5—C33—C31	112.4 (3)
C7—C8—H8	119.5	C33—C34—C35	120.1 (4)
C9—C8—H8	119.5	C33—C34—H34	120.0
C10—C9—C8	118.0 (4)	C35—C34—H34	120.0
C10—C9—H9	121.0	C34—C35—C36	118.3 (4)
C8—C9—H9	121.0	C34—C35—H35	120.8
C11—C10—C9	122.3 (4)	C36—C35—H35	120.8
C11—C10—C12	114.5 (3)	C35—C36—C37	121.1 (4)
C9—C10—C12	123.1 (4)	C35—C36—H36	119.4
C10—C11—C6	117.4 (4)	C37—C36—H36	119.4
C10—C11—Pt1	115.3 (3)	N5—C37—C36	120.1 (4)
C6—C11—Pt1	126.4 (3)	N5—C37—C38	119.0 (3)
C13—C12—N2	121.7 (4)	C36—C37—C38	120.3 (3)
C13—C12—C10	125.7 (4)	C39—C38—C42	114.6 (3)
N2—C12—C10	112.5 (3)	C39—C38—C37	124.0 (3)
C12—C13—C14	120.2 (4)	C42—C38—C37	121.1 (3)
C12—C13—H13	119.9	N6—C39—F3	114.3 (4)
C14—C13—H13	119.9	N6—C39—C38	126.7 (4)
C13—C14—C15	118.6 (4)	F3—C39—C38	118.9 (4)
C13—C14—H14	120.7	N6—C40—F4	115.0 (4)
C15—C14—H14	120.7	N6—C40—C41	126.4 (4)
C14—C15—C16	120.9 (4)	F4—C40—C41	118.6 (4)
C14—C15—H15	119.6	C40—C41—C42	115.7 (4)
C16—C15—H15	119.6	C40—C41—H41	122.1
N2—C16—C15	120.5 (4)	C42—C41—H41	122.1
N2—C16—C17	118.4 (3)	C41—C42—C38	121.2 (4)
C15—C16—C17	120.5 (3)	C41—C42—H42	119.4
C21—C17—C18	114.7 (4)	C38—C42—H42	119.4
			
C5—N1—C1—C2	−1.9 (6)	C26—N4—C22—C23	−0.8 (6)
Pt1—N1—C1—C2	173.2 (3)	Pt2—N4—C22—C23	174.2 (3)
N1—C1—C2—C3	0.3 (7)	N4—C22—C23—C24	−0.1 (7)
C1—C2—C3—C4	0.2 (7)	C22—C23—C24—C25	0.5 (8)
C2—C3—C4—C5	1.0 (8)	C23—C24—C25—C26	0.0 (8)
C1—N1—C5—O1	−177.3 (4)	C22—N4—C26—O2	−176.9 (4)
Pt1—N1—C5—O1	8.0 (6)	Pt2—N4—C26—O2	8.5 (6)
C1—N1—C5—C4	3.2 (6)	C22—N4—C26—C25	1.3 (6)
Pt1—N1—C5—C4	−171.6 (4)	Pt2—N4—C26—C25	−173.3 (4)
C6—O1—C5—N1	10.8 (7)	C27—O2—C26—N4	6.4 (7)
C6—O1—C5—C4	−169.6 (4)	C27—O2—C26—C25	−172.0 (4)
C3—C4—C5—N1	−2.9 (8)	C24—C25—C26—N4	−0.9 (8)
C3—C4—C5—O1	177.5 (5)	C24—C25—C26—O2	177.5 (5)
C5—O1—C6—C7	161.3 (4)	C26—O2—C27—C28	165.0 (4)
C5—O1—C6—C11	−21.3 (7)	C26—O2—C27—C32	−16.3 (7)
O1—C6—C7—C8	−178.6 (4)	O2—C27—C28—C29	−177.4 (4)
C11—C6—C7—C8	4.0 (7)	C32—C27—C28—C29	3.9 (7)
C6—C7—C8—C9	−2.0 (7)	C27—C28—C29—C30	−1.5 (7)
C7—C8—C9—C10	−0.8 (7)	C28—C29—C30—C31	−1.5 (7)
C8—C9—C10—C11	1.7 (6)	C29—C30—C31—C32	2.1 (6)
C8—C9—C10—C12	−176.0 (4)	C29—C30—C31—C33	−176.0 (4)
C9—C10—C11—C6	0.2 (6)	C30—C31—C32—C27	0.1 (6)
C12—C10—C11—C6	178.1 (3)	C33—C31—C32—C27	178.4 (3)
C9—C10—C11—Pt1	169.8 (3)	C30—C31—C32—Pt2	169.9 (3)
C12—C10—C11—Pt1	−12.4 (4)	C33—C31—C32—Pt2	−11.8 (4)
O1—C6—C11—C10	179.7 (4)	C28—C27—C32—C31	−3.2 (6)
C7—C6—C11—C10	−3.1 (6)	O2—C27—C32—C31	178.2 (4)
O1—C6—C11—Pt1	11.4 (6)	C28—C27—C32—Pt2	−171.7 (3)
C7—C6—C11—Pt1	−171.3 (3)	O2—C27—C32—Pt2	9.7 (6)
C16—N2—C12—C13	7.8 (5)	C37—N5—C33—C34	8.4 (5)
Pt1—N2—C12—C13	−167.8 (3)	Pt2—N5—C33—C34	−167.7 (3)
C16—N2—C12—C10	−169.1 (3)	C37—N5—C33—C31	−169.4 (3)
Pt1—N2—C12—C10	15.3 (4)	Pt2—N5—C33—C31	14.5 (4)
C11—C10—C12—C13	−179.3 (4)	C32—C31—C33—C34	179.9 (4)
C9—C10—C12—C13	−1.5 (6)	C30—C31—C33—C34	−1.8 (6)
C11—C10—C12—N2	−2.5 (5)	C32—C31—C33—N5	−2.3 (5)
C9—C10—C12—N2	175.3 (3)	C30—C31—C33—N5	175.9 (3)
N2—C12—C13—C14	−1.3 (6)	N5—C33—C34—C35	−2.4 (6)
C10—C12—C13—C14	175.2 (4)	C31—C33—C34—C35	175.1 (4)
C12—C13—C14—C15	−4.0 (6)	C33—C34—C35—C36	−3.1 (6)
C13—C14—C15—C16	2.6 (6)	C34—C35—C36—C37	2.4 (6)
C12—N2—C16—C15	−9.2 (5)	C33—N5—C37—C36	−8.9 (5)
Pt1—N2—C16—C15	165.6 (3)	Pt2—N5—C37—C36	166.4 (3)
C12—N2—C16—C17	162.7 (3)	C33—N5—C37—C38	162.7 (3)
Pt1—N2—C16—C17	−22.5 (5)	Pt2—N5—C37—C38	−21.9 (5)
C14—C15—C16—N2	4.2 (6)	C35—C36—C37—N5	3.8 (6)
C14—C15—C16—C17	−167.5 (4)	C35—C36—C37—C38	−167.8 (4)
N2—C16—C17—C21	−45.1 (5)	N5—C37—C38—C39	142.1 (4)
C15—C16—C17—C21	126.7 (4)	C36—C37—C38—C39	−46.3 (5)
N2—C16—C17—C18	141.4 (4)	N5—C37—C38—C42	−44.8 (5)
C15—C16—C17—C18	−46.8 (5)	C36—C37—C38—C42	126.8 (4)
C19—N3—C18—F1	−178.8 (4)	C40—N6—C39—F3	−178.8 (3)
C19—N3—C18—C17	1.0 (6)	C40—N6—C39—C38	0.3 (6)
C21—C17—C18—N3	−2.0 (6)	C42—C38—C39—N6	−1.7 (6)
C16—C17—C18—N3	171.9 (4)	C37—C38—C39—N6	171.7 (4)
C21—C17—C18—F1	177.7 (3)	C42—C38—C39—F3	177.4 (3)
C16—C17—C18—F1	−8.4 (6)	C37—C38—C39—F3	−9.2 (5)
C18—N3—C19—F2	−176.8 (3)	C39—N6—C40—F4	−177.1 (3)
C18—N3—C19—C20	1.0 (6)	C39—N6—C40—C41	2.3 (6)
N3—C19—C20—C21	−1.7 (6)	N6—C40—C41—C42	−3.1 (6)
F2—C19—C20—C21	176.1 (3)	F4—C40—C41—C42	176.4 (3)
C19—C20—C21—C17	0.4 (5)	C40—C41—C42—C38	1.3 (5)
C18—C17—C21—C20	1.2 (5)	C39—C38—C42—C41	0.8 (5)
C16—C17—C21—C20	−172.8 (3)	C37—C38—C42—C41	−172.9 (3)

### Hydrogen-bond geometry (Å, º)

**Table:**

*D*—H···*A*	*D*—H	H···*A*	*D*···*A*	*D*—H···*A*
C1—H1···Cl1	0.95	2.46	3.175 (4)	132
C3—H3···F1^i^	0.95	2.53	3.474 (5)	172
C15—H15···F1	0.95	2.56	2.989 (5)	108
C22—H22···Cl2	0.95	2.45	3.170 (4)	132
C23—H23···Cl2^ii^	0.95	2.82	3.540 (5)	133
C24—H24···F3^iii^	0.95	2.50	3.443 (5)	172
C36—H36···F3	0.95	2.55	2.975 (5)	108
C41—H41···Cl1	0.95	2.79	3.684 (4)	158

Symmetry codes: (i) *x*, *y*+1, *z*; (ii) −*x*+2, −*y*+1, −*z*; (iii) *x*−1, *y*, *z*.
